# Roadside Drug Testing Approaches

**DOI:** 10.3390/molecules26113291

**Published:** 2021-05-29

**Authors:** Manal A. Alhefeiti, James Barker, Iltaf Shah

**Affiliations:** 1 Department of Chemistry, College of Science, United Arab Emirates University, Al Ain P.O. Box 15551, United Arab Emirates; 2 School of Life Sciences, Pharmacy and Chemistry, Kingston University, Surrey KT1 2EE, UK; j.barker@kingston.ac.uk

**Keywords:** LC-MS/MS, roadside testing, drug testing, drugs of abuse, substance abuse

## Abstract

The purpose of this review is to present an overview of roadside drug testing, driving enforcement, and drunk/drug driving detection around the world. Drunk and drug driving is a severe problem, not only in the UAE, but also around the world. This has important implications for road safety as drunk or drug driving may increase the chances of a driver’s involvement in a road crash when compared to a drug-free driver. Recently, due to increases in drug-impaired drivers’ crash involvement, many mobile roadside drug testing devices have been introduced to the market. These devices use oral fluid, urine or blood matrices. These are on-the-spot tests, which are easy to use and are applied by law enforcement agencies and the public. Law enforcement agencies most commonly use oral fluid to detect the presence of illicit drugs in drivers. This review discusses all the available devices in the market used by the authorities. It also describes the type of drugs widely abused by drivers along with behavioral testing methods. The different types of matrices used for roadside drug testing are also evaluated. Sample collection, storage, and pre-treatment methods are discussed, followed by the confirmatory analysis of positive samples. This article will significantly help law enforcement agencies compare and evaluate all the reliable roadside testing devices and new emerging confirmatory devices available to them in the market. This will help them make an informed decision on which device to adapt to their individual needs.

## 1. Introduction

In Dubai alone, 14.33% of crashes are caused by drivers under the influence of alcohol. In the Middle East and worldwide, drug abuse is a growing problem for authorities that requires significant action [1].

The risk of driving under the influence of drugs does not only harm the drivers but can cause injury to others as well [2]. The consequences of legal and illegal psychotropic drugs concerning a reduced capacity to drive have been widely recorded. Opioids, analgesics, benzodiazepines, antidepressants, cannabinoids, cocaine, and amphetamines are examples of psychoactive drugs that can cause these impaired effects [3]. Statistics obtained from a recent project funded by the EU called “Driving Under the Influence of Drugs Alcohol and Medicines (DRUID]” show several interesting facts. Firstly, around 2% of drivers in Europe have admitted to using recreational drugs when driving. Secondly, it was found that 28 to 53% of the drivers who have been seriously injured in accidents were under the influence of a psychoactive drug (mostly alcohol, medicinal, or recreational drugs).

Moreover, in Belgium, it was found that 5% of the drivers that were seriously injured in accidents were under the influence of a single illegal drug, either high levels of cannabis or a mixture of cannabis with alcohol found in 1% of the drivers [4,5]. Due to these findings in the EU, law enforcement agencies have been putting greater emphasis on controlling driving under the influence of drugs. Driving under the influence is also a big problem in the USA [6,7].

Roadside drug testing is one way police are trying to curb drug misuse [8,9]. The police will use on-the-spot drug screening techniques such as drug wipes, oral fluid screens or breath tests to ascertain drug misuse. If found above the cut-off limit, these samples will then be sent for confirmation in blood, urine and saliva using more advanced and time-consuming techniques such as GC-MS, LC-MS, etc. Recently, hair analysis techniques have been used for confirmation of long-term drug abuse [10]. This review will focus on setting out existing preventative approaches to drug abuse and secondly on outlining the developments and advances in on-the-spot drug testing technologies along with confirmatory analysis of positive samples.

### Medical Consequences of Drug Abuse

Drug and alcohol abuse causes different side effects on the human body. For example, stimulants such as cocaine and methamphetamine (also known as meth, speed, or ice) cause a constriction of the body’s blood vessels along with increased heart rate. In those individuals that are more pre-disposed to illness, these two effects together can result in stroke and cardiac arrhythmias. Other severe effects of methamphetamine include hyperthermia, hypertension, chest pain, and convulsions [11,12]. The use of cocaine causes an increase in heart rate and blood pressure, along with an increase in blood clots, which can lead to the risk of a heart attack. Research carried out by the National Institute of Drug Abuse (NIDA) reveals that prolonged cocaine use is related to left ventricular dysfunction and high coronary calcium deposits in African Americans. These patients were co-diagnosed with HIV, and it was found that cocaine use causes the spread of HIV into brain cells, leading to HIV encephalopathy [13]. MDMA (methylenedioxymethamphetamine), also recognized as ecstasy, is a popular club drug that is often wrongly thought to be safe by those taking it. Recently, it has been known to cause malignant hyperthermia, permanent kidney and brain–serotonin–nerve-fiber impairment and death [14,15,16]. Focal glomerulosclerosis, which can be a fatal renal kidney disorder, is caused by heroin use. The use of opiates can cause numerous health problems such as nausea, dental, orofacial and renal problems [16,17]. Lowered heart rate and blood pressure, which lead to aggressiveness, are the consequence of taking phencyclidine (PCP), also known as angel dust [13]. Cannabinoids are the most widely abused drug worldwide and are also thought to be the most harmless drug used by individuals. The side effects of the use of cannabinoids vary from loss of memory, problems with cognition, and even lung cancer in those that use it frequently [18]. Drug users who inject and partake in these drugs to engage in thoughtless sexual activity are at a higher risk of attaining sexually transmitted or blood-borne infections or both. Some of these infections can be fatal, including HIV, AIDS, hepatitis, herpes, endocarditis, and other sexually transmitted diseases. From this, we can say that police controls on the roadside are for the benefit of maintaining public health (as well as transgression) [13,16].

## 2. Drug Effects on Drivers

In this section, various drug classes, their association with each other, their levels in biological specimens and their effects on driving capability will be discussed.

### 2.1. Cannabinoids

Cannabinoids refer to the chemical compounds distinctive of cannabis plants, with the chief pharmacologically active constituent being delta 9 -tetrahydrocannabinol (THC). THC is a depressant of the central nervous system. It can cause numerous problematic effects such as dizziness, confusion, hallucinations, speech and vision problems, ataxia, euphoria, lack of strength, and fatigue [19]. Tests have shown that if an individual takes just one THC dose, either by ingestion or smoking, it can have a substantial adverse effect on psychomotor performance. This is seen for a duration of 3 h in those that are driving, and up to 24 h in lab study volunteers [20,21].

### 2.2. Benzodiazepines

Diazepam and alprazolam are types of benzodiazepines that can be obtained by prescription. When abused, they depress the central nervous system (CNS), which can cause side effects such as confusion, weariness, dizziness, and drowsiness [22]. Those who are prescribed to take these drugs are advised not to carry out activities that need mental attentiveness or to take any other CNS depressant drug or alcohol. Driving simulation studies have also shown that benzodiazepines can substantially impair driving capabilities [20]. Just one dose of diazepam can cause a lack of ability to drive within a single lane, slow the reaction times, reduce attention, decrease the capability to multi-task, have adverse effects on cognition, and can result in higher fatigue levels. When used along with low levels of alcohol, they can further impair driving capability [23].

### 2.3. Opiates

Morphine, codeine, and heroin are the most common types of opiates. Opioids are CNS depressants that can cause lethargy, confusion, ataxia, dizziness, drowsiness, visual impairment and weakness [24]. For those opiates that are used for medicinal purposes, manufacturers have warned not to use them when carrying out possibly dangerous tasks. This is due to the impairment that may be caused to an individual’s physical and mental capabilities [25]. Morphine ingested orally or by intravenous and intramuscular delivery has been shown to cause sedation and reduce the psychomotor capability for up to 4 h. This was the case for a single dose in both healthy volunteers and past addicts. In laboratory research, this caused impairment for up to 36 h after numerous doses [20,22].

### 2.4. Methadone

Methadone is another CNS depressant known to cause disorientation, weakness, drowsiness, light-headedness and visual impairment [26,27]. Studies have shown that just one dose of methadone in volunteers who have not taken it previously can cause substantial impairment in driving capacity. For those that take it long term in small quantities, the psychomotor and cognitive effects are negligible as long as no other drugs are being used along with it [20].

### 2.5. Cocaine

Cocaine has numerous effects as a CNS stimulant, including insomnia, euphoria, fatigue, dyskinesia, dizziness, tremors, and dysphoria [28,29]. Prolonged use can cause changes in personality, psychosis, hyperactivity, and tetchiness [20]. Substantial impairment in driving capacity has been seen by drivers under the influence who have been caught speeding, causing accidents, not paying attention and losing control of their vehicle. Tiredness, depression and insomnia are all effects of cocaine that wear off [20].

### 2.6. Amphetamines, Methamphetamines and Ecstasy (MDMA)

Various adverse effects caused by CNS stimulants such as amphetamine and methamphetamine are restlessness, disorientation, agitation, nervousness, insomnia, tremor, agitation, dizziness, euphoria, fast speech, staggering, dyskinesia, and dysphoria in users. Prolonged use can lead to changes in personality, psychosis, and hyperactivity [30,31]. Those under the influence of these drugs have been seen to drive unpredictably, overspeed, and cause collisions. The lack of driving capacity is due to being confused, unfocused, overactive, excited, having decreased cognition, withdrawal symptoms, tiredness, and hypersomnolence [30]. Studies related to driving impairment caused by MDMA/ecstasy have shown cognitive adverse effects on vehicle control, levels of risk, and cognitive and information processing impairment. MDMA is a weaker CNS stimulant, which causes sensory instabilities, nausea, ataxia, restlessness, tremor, and muscular rigidity [20].

### 2.7. Prescription Drugs

The risk of collision or a driving incident is lower with prescription drugs, as these are not taken at high levels. In addition to tolerance building, they could also produce significant effects when used continuously [2]. This phenomenon is compared to the use of illegal drugs or drug overdose against what is prescribed by a doctor. Recent research has shown that taking multiple CNS prescription drugs does not diminish the risk of impairment in individuals over the age of 45 [20].

### 2.8. Novel Psychoactive Substances

Novel psychoactive substances (NPS) have been an important part of clinical and forensic toxicology for over 100 years. This began with the introduction of several new drugs such as heroin, 3,4-methylenedioxymethamphetamine (MDMA), lysergic acid diethylamide (LSD), and γ-hydroxybutyric acid (GHB). However, after the appearance of synthetic cannabinoids at the start of this century, there was a rapid increase in the number of synthetic cathinones, benzodiazepines and opioids, which now count in the hundreds. Toxicology laboratories previously focused on a rather narrow range of compounds, including amphetamines, cocaine, opioids, cannabinoids, salicylate, antidepressants and acetaminophen. Now, potent fentanyl derivatives are mixed with heroin or used on their own and could kill drug users very quickly. Toxicology laboratories have difficulty in detecting potent psychoactive drug analogues that are present in the blood for only a short amount of time due to unknown urinary metabolites and a lack of available reference standards [32].

Despite the increasing range of NPS and also the incontrovertible fact that fatal and acute intoxication cases are already attributed to current novel psychoactive compounds, this development seems to be significantly underestimated, mainly because of the substantial lack of comprehensive screening strategies for the detection of NPS in biological samples. Continuous addition to the latest entries to the family of NPS, along with the wide physico-chemical properties of substances of this class, make their detection in biological specimens very challenging in clinical and forensic toxicology. The development of analytical methods for the detection of the latter substances in either conventional and non-conventional samples is of huge importance to drug metabolism research and also for associating intake with clinical outcomes and ultimate intoxication symptoms [33].

## 3. Roadside Testing

### 3.1. Physical/Behavioral Testing

A standardized approach for physical drug testing includes: (a) observing physical signs and (b) attention distributive tests [5]. Physical signs include the appearance of the eyes (glowy, blurred, or irregular) and shaking or trembling in any part of the body. The attention tests include: (i) the one-leg stand test, (ii) the Romberg test, (iii) the walk and turn test, and (iv) the finger to nose test. A positive test battery consists of a minimum of one of the physical signs along with one of the attention distributive tests [34]. A positive test result consists of the signs from any of the following categories, which are the (a) eyes (shiny, hazy, red/bloodshot, contracted pupils), (b) face (dehydrated lips, grating teeth, snorting repetitively), (c) behavior (anxious, violent, confused), (d) state of mind (changing mood, overly excited), (e) language (mumbling, long-winded, repetition of words), (f) walk (not balanced), and (g) others (shaking, sweating, reflexes that are overly fast or slow) [5].

### 3.2. On-Site Screening Testing

**Figure 1:**
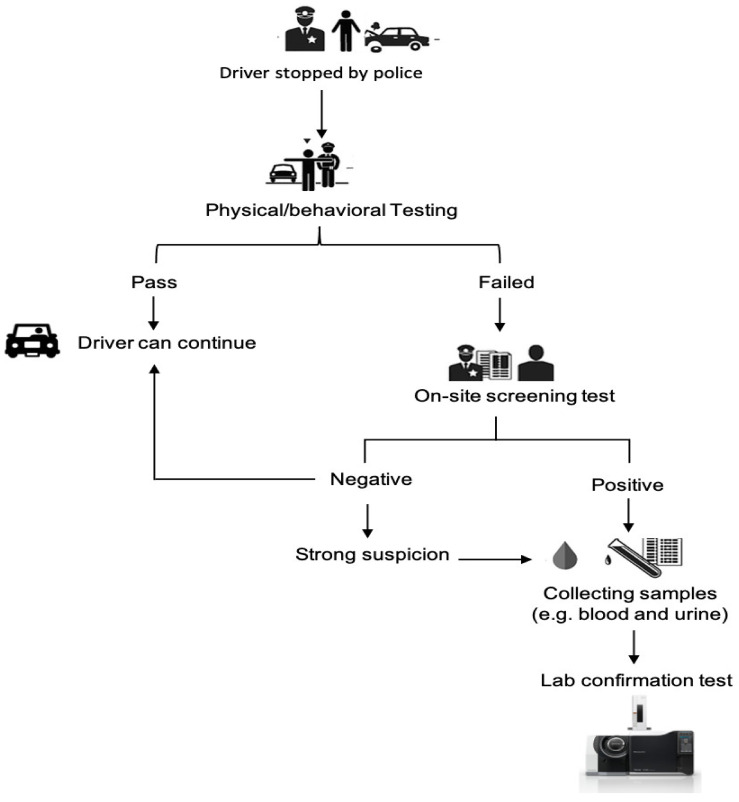
Flow diagram of legal procedures in the UAE.

Over the last 20 years, there has been increased research interest in developing the most reliable and appropriate roadside drug testing devices. There are many roadside drug testing devices in use by law enforcement agencies. The detected roadside drugs are later verified using confirmatory tests [5]. On-site screening technology allows for multiple drugs to be tested simultaneously with the use of a single body fluid sample. These tests are designed to be simple, with only a few steps, and are disposable and hence only used once [8]. Other tests may have more steps, some of which are timed. Immunoassay tests work by initially collecting a body fluid sample taken in a collector that is either added immediately to the testing cartridge or, in some cases, a buffer is added. The sample is added to the immunoassay strip; most tests run at the surrounding environment temperature, whereas others need to be incubated to exact temperatures [5,35]. Dried blood spots could also be considered as an on-site blood collection technique [36,37]. Figure 1 illustrates the legal procedures followed by law enforcement agencies in the UAE.

The cut-off limits for drugs differ from one country to another, as do the collection times for the body fluid sample and time to carry out the actual test. Some police forces use electronic devices, whereas others use non-electronic devices, where the test results need to be visually read and recorded. These parameters depend on what the force wants to achieve from the device given their budget [3,35,38,39].

## 4. Specimen Types

**Figure 2:**
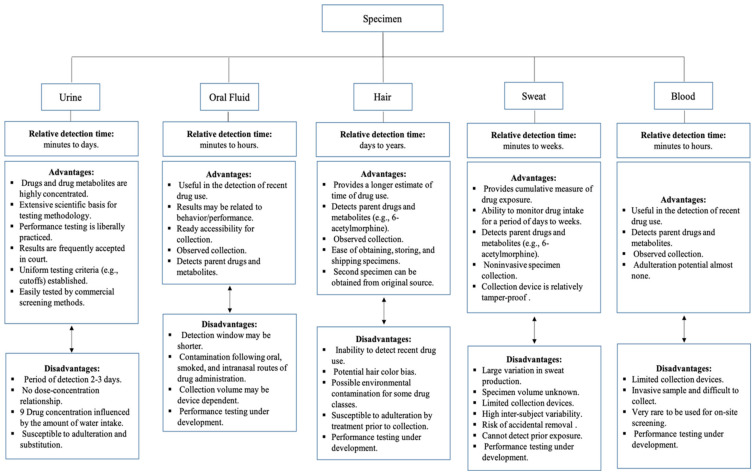
Advantages and disadvantages of various specimens [9].

What body fluid specimen is taken usually depends on how easy it is to obtain the sample and the analytical and testing implications of the results. Usually, roadside testing involves collecting oral fluid, urine, blood, or breath [9,20,38]. There are numerous benefits and drawbacks to each type of sample type when testing in a roadside setting, and these are depicted in Figure 2 [9]. Usually, blood and urine samples are collected at the police station instead of at the roadside.

### 4.1. On-Site Drug Testing Devices

Numerous types of on-site drug testing systems will be discussed in this review. Table 1 summarizes information about some of the devices used for on-site drug testing. Some of the advantages and disadvantages of these devices are listed in Table 2.

### 4.2. Securetec Drugwipe

**Figure 3:**
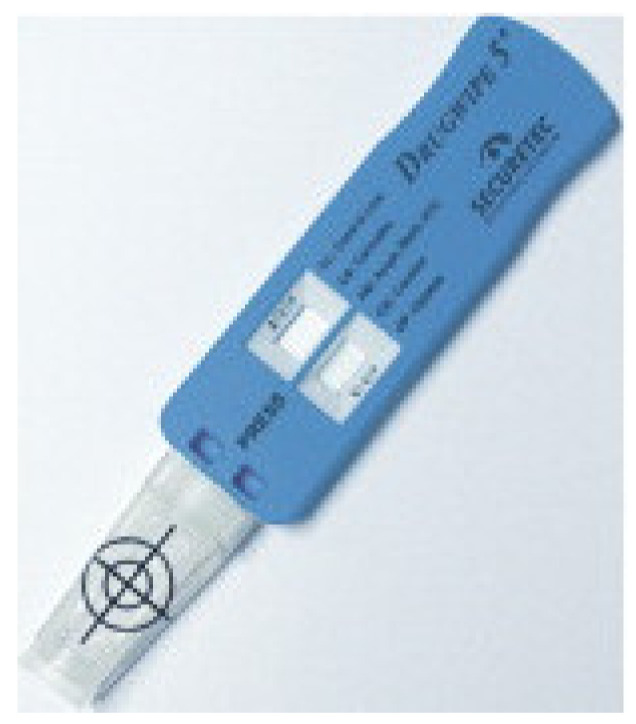
Saliva drug test Securetec DrugWipe device (reproduced with permission from Elsevier, Ref. [62]).

Figure 3 shows the saliva drug test Securetec DrugWipe device, which has a removable oral fluid collector that is used to take the driver’s sample and then placed back into the test device [3]. A color indicator is used to show that saliva has been successfully collected into the collector, and this process takes five seconds. A buffer is required for this test device and is intrinsic to the device. It is used in the form of a capsule, which, when crushed, discharges the buffer into the immunoassay strip pushing the sample into the test area. The device needs to be kept upright for 15 s after crushing the buffer capsule, and a sliding cover must be used to cover the test area. At this point, the device should be laid and left flat on a level surface for a couple of minutes. Following this process, the result will be available and could be read to show the drug’s presence or absence based on the red lines that appear in the corresponding boxes. The principle of this device can be summarized as follows: once the sample collector transfers the sample to the test strips, which contain drug-specific antibodies, positive samples that contain certain drugs will bind to their antibodies and a red line will be shown. The test begins as soon as the test strip is immersed in a buffer. The buffer helps the drugs that are bound to the antibodies to migrate to the test line. The red test line is evaluated visually [4,21,38,59,60].

### 4.3. Draeger DrugTest 5000 Analyzer

**Figure 4:**
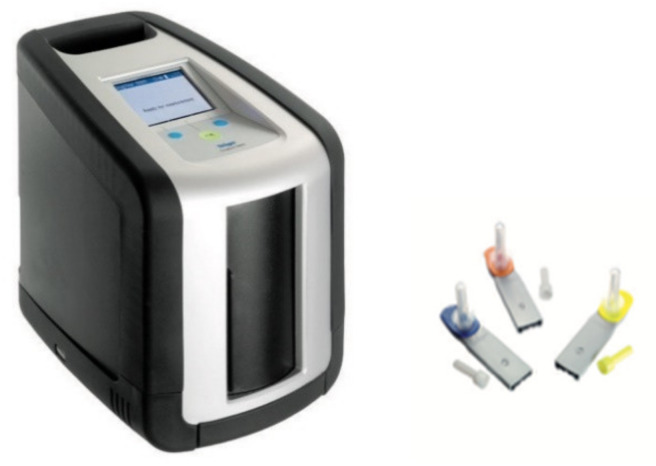
Dräger DrugTest^®^ 5000 device (reproduced with permission from Elsevier, Ref. [62]).

This analyzer comprises a cassette with both the sample collector and immunoassay present, and a buffer is also required for this test [3]. Saliva is collected in the collector, after which a one-minute wait time is needed to ascertain whether enough oral fluid sample has been provided (indicator will turn blue if it has) as depicted in Figure 4 [62]. If not enough sample is obtained, there is a three-minute window for a further fluid sample to be provided, after which the test cassette is placed in the lower section of the analyzer and the buffer cartridge in the upper section. Once everything is in place, the analyzer door is closed, and this starts the analysis process automatically. Those using the cassette can check their validity date, the kit batch number, and drugs tested on the analyzer. Draeger 5000 can determine drugs in saliva and also on surfaces. This device weighs around 6 kg and costs around EUR 4000. The results are obtained after eight minutes in the form of the specified drug identified or not identified [3,20].

### 4.4. Alere DDS2

**Figure 5:**
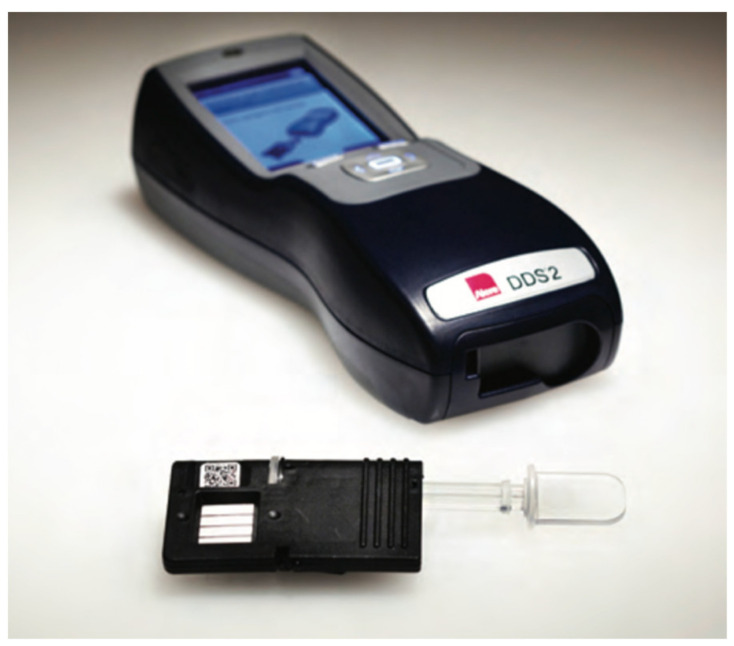
ALERE DDS^®^2 mobile test system (reproduced with permission from *Journal of Analytical Toxicology* [47]).

This mobile test system has a test cartridge and sample collector, which need to be inserted into the device [20]. Initially, the test cartridge has to be placed in the analyzer, which prompts the user to obtain an oral fluid sample from the driver within two minutes. Once this is carried out, the sample collector can be placed within the test cartridge, which is already in the analyzer (Figure 5) [37]. The test cartridge serves the purpose of holding the collector and contains dried agents, buffer, and immunoassay test strips. A duration of five minutes is required for sample analysis. This analyzer can be used to test five or six different drugs (amphetamine, benzodiazepine, THC, cocaine metabolite, methamphetamine, and opiates) dependent upon the test cartridges purchased [3,20]. This device weighs around 680 g and costs around EUR 6000.

### 4.5. Mavand Rapid STAT

**Figure 6:**
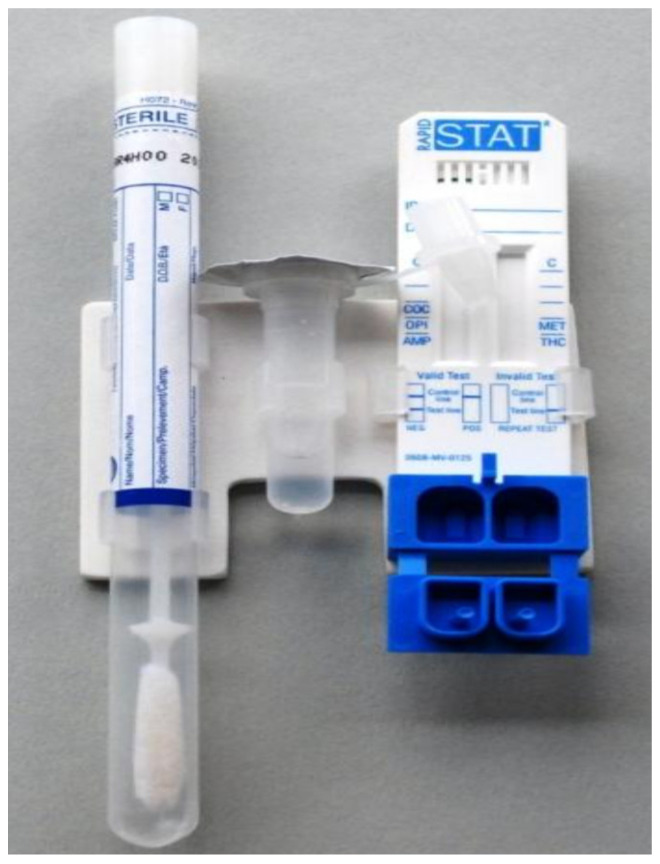
Mavand Rapid STAT^®^ saliva multi-drug test system (reproduced with permission from Elsevier [62]).

The rapid STAT saliva multi-drug test system, manufactured by Mavand, provides a disposable test system that is non-digital and made up of a sample collector, buffer bottle, and test cassette joined in a one-hand-clip system (Figure 6) [62]. This device can test anywhere from two to seven drugs, including amphetamines, benzodiazepines, cocaine, methadone, methamphetamine, MDMA, opiates, and THC. The device’s test cassette has two reaction compartments and immunoassay test strips. The production of saliva is stimulated by an unknown compound in the sample collector [36]. Once the oral fluid is collected, the collector sponge is added to the buffer bottle. This solution of both saliva and buffer is added to the reaction compartments that contain antibodies. There is a four-minute wait time to allow for the incubation process to happen, after which the solution is added to the test strips, and analysis takes place. After eight minutes, the results should be available and read visually and interpreted depending upon which lines are present against the cartridge’s drug name [3,20].

### 4.6. iScreen Test Device

**Figure 7:**
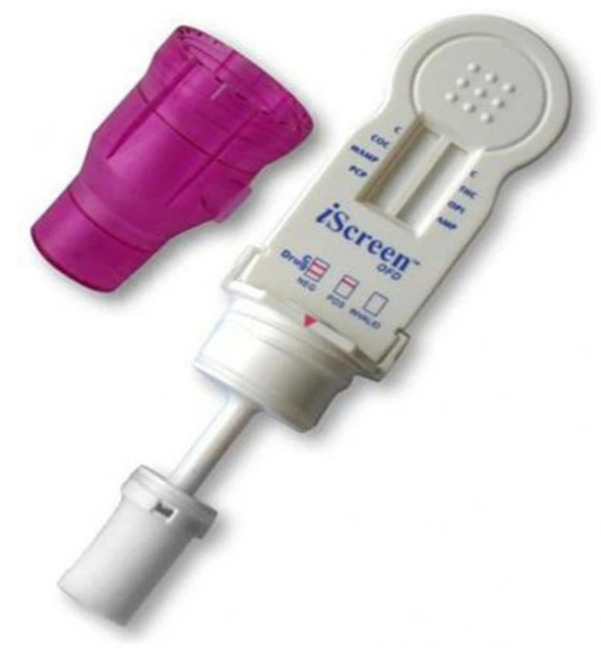
iScreen Test Device [48].

The manufacturer of this device is Acon Laboratories Inc. 10125 Mesa Rim Road, San Diego, CA 92121, USA, (Figure 7). It is used to test for cocaine (COC), marijuana (THC), methamphetamine (Meth), amphetamine (AMP), opiates (OPI), and phencyclidine (PCP). Saliva is the required matrix for this test. To collect the sample, a sponge is inserted into the person’s mouth to collect an oral fluid sample (duration: 3 min). The collector is then placed into the device clockwise until engaged, and a one-minute wait time is applied. After this, the collection chamber is rotated clockwise, and a wait time is required for the result. The analysis process takes around 10 min for the result [48]. This test device (Oral Fluid) is an immunoassay based on the principle of competitive binding. Drugs that may be present in the oral fluid specimen compete against their respective drug conjugate for binding sites on their specific antibody. During testing, a portion of the oral fluid specimen migrates upward by capillary action. If present in the oral fluid specimen below its cut-off concentration, a drug will not saturate the binding sites of its specific antibody. The antibody will then react with the drug–protein conjugate, and a visible colored line will show up in the test line region of the specific drug strip. The presence of a drug above the cut-off concentration in the oral fluid specimen will saturate all the binding sites of the antibody. Therefore, the colored [48] iScreen systems can also determine drugs not only in saliva but also on surfaces, similar to the Draeger 5000.

### 4.7. OraLab

**Figure 8:**
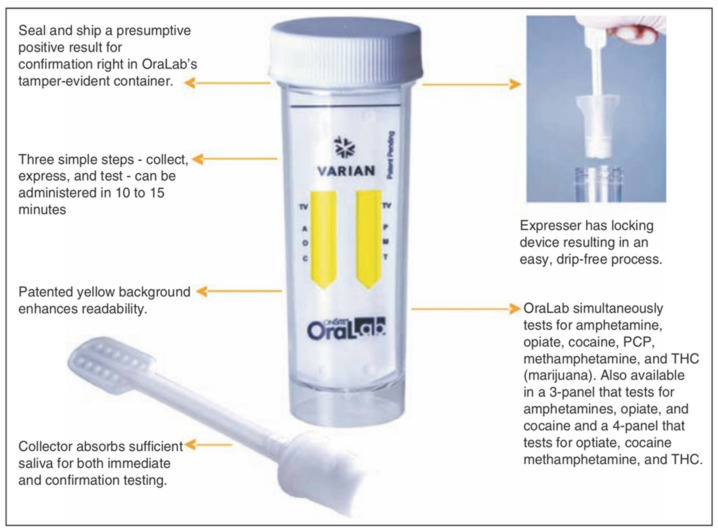
Varian OraLab (reproduced with permission from Springer [51]).

Varian Inc. manufactures the OraLab saliva drug test system to test for cocaine, morphine, amphetamines, methamphetamine, benzodiazepines, phencyclidine, and THC. It has a shelf-life of around 12 months and can be stored at room temperature [50]. The principle of this system is competitive inhibition. An immobilized drug conjugate along with coated antibody (red-labelled) microparticles are linked onto a membrane. Once the collector foam is introduced into the tube, the oral fluid sample is absorbed by the test card’s membrane. Drug conjugates will start competing against drugs in the sample for interaction with the coated antibody microparticles, resulting in a clear line beside the corresponding drug’s name. The result takes about 10 to 15 min to be produced [49]. Figure 8 shows the OraLab test system [50,51].

### 4.8. AconLabs Oral Fluid Drug Screen

**Figure 9:**
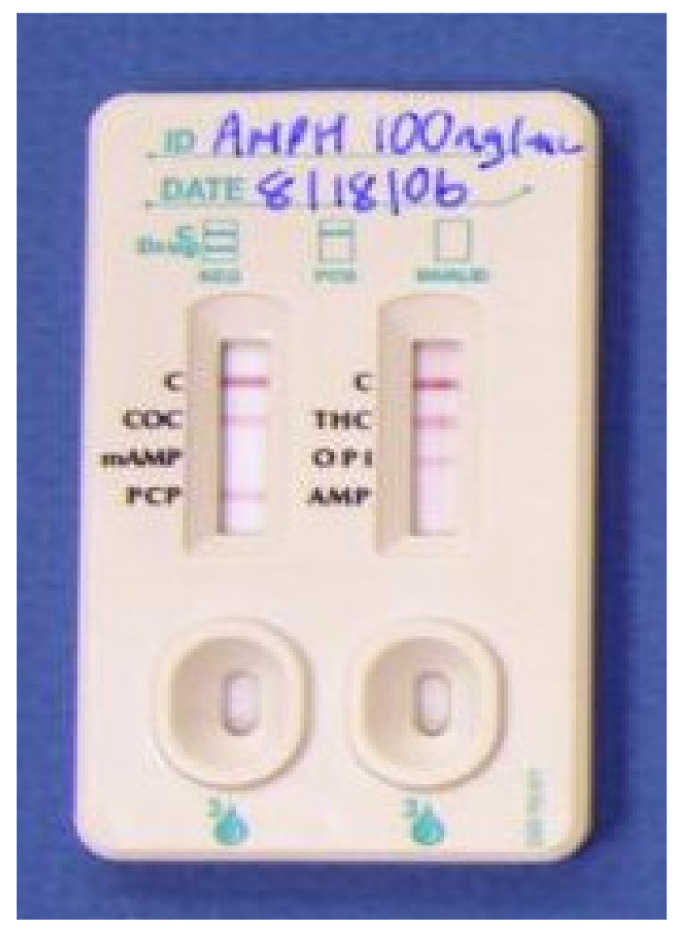
AconLabs Oral Fluid Drug Screen device [48].

AconLabs Oral Fluid Drug Screen is a saliva-based test covering six ranges of drugs (cocaine, cannabis, amphetamines, methamphetamines, opiates, and phencyclidine). A saliva specimen is collected by inserting a sponge in the mouth (for three minutes). This is followed by opening the upper part of the collection vial and introducing the sponge. In order to extract the oral fluid from the sample sponge, the sponge is pushed down into the container. Then, the upper part of the collection vial is closed and the cap exposing the dropper is removed. The last step requires squeezing the end chamber of the collection vial and adding around three drops to the sample well cartridge. The results are released within 10 min. The test system has to be kept on a clean and level surface (Figure 9) [48,50].

### 4.9. OralStat

**Figure 10:**
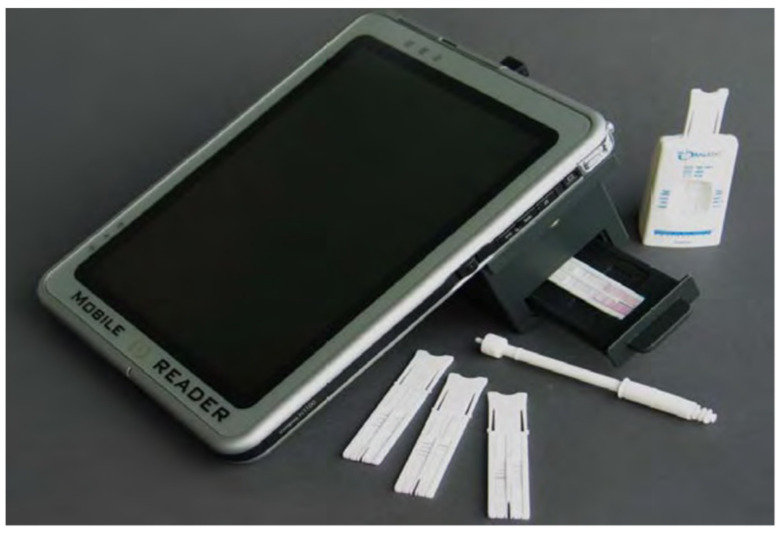
OralStat device [50].

This system (Figure 10) works only with saliva specimens and can detect several groups of drugs such as methamphetamines, amphetamines, cocaine, opiates, cannabis, benzodiazepines, phencyclidine, and methadone. This testing device is based on colloidal gold technology. The device functions as follows: 1) the label is taken off and the test slide verified whether it is in the upward position. 2) The collection swab handle is placed in every one of the “A” wells, pressed down entirely and shaken from side to side for a minimum of three seconds. 3) In between the gum and cheek on each side of the mouth, a sponge is pressed for a minimum of 60 s until the sponge completely expands. 4) The sponge is placed in the “B” well and forced downwards until it is set in place. This is kept in place for at least five seconds. 5) Then, for a minimum of three seconds, it is stirred from side to side. The test is left for eight minutes. The tabs are pressed in and the test slide pushed downwards. 6) After two to four minutes, the results are ready [50].

### 4.10. Oratect^®^ II

This is a saliva-based test that produces a result within eight minutes, and a single step is required for the oral fluid collection and testing. The administrator aids in handling the elimination of the bodily fluid [50]. The sample volume needed in order to accommodate donors with dry mouth is about 0.5 mL. Six drugs, including amphetamine, marijuana (THC), cocaine, benzodiazepines, opiates, phencyclidine, and methamphetamine, can be detected by Oratect^®^II. In the mouth’s cavity, parent THC is present instead of THCCOOH, which is the carboxylic acid metabolite; thus, to have a longer window for detection, the cut-off concentration of the final concentration is decreased to 40 ng/mL. The optimal storage temperature is around room temperature, which ranges between 59 and 86 °F (15 and 30 °C).

**Figure 11:**
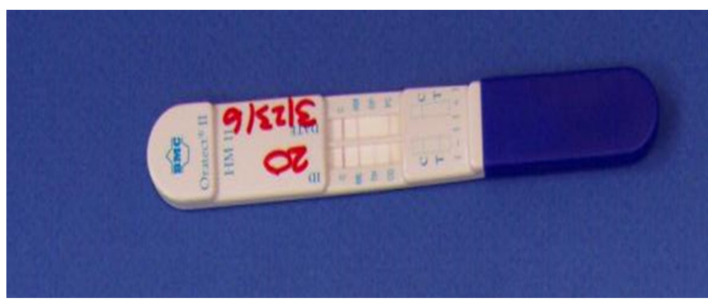
Branan Medical Oratect^®^ saliva testing system [48].

Drugs in the oral fluid compete for the low number of binding sites of the antibody on colored colloidal gold antibody conjugate with the derivatives of the drugs that are immobilized on the membrane. This whole process is a competitive immunoassay procedure, which is the build concept of this device. The collection pad is used to obtain the saliva, and it moves across the membrane during the process of testing, which can be seen by the change in the blue lines (as shown in Figure 11). The colored colloidal gold antibody conjugate attaches to the derivatives of the drug on the membrane, and this leads to the formation of bands that are visible at the particular region of testing. This happens if there is no presence of the drug in the oral fluid. A negative result is denoted by the formation of colored bands at the region of testing. A positive test result, on the other hand, is denoted by the absence of a band of color at the region of testing. However, whatever the case, if the test has been conducted properly, it is indicated by the color bands’ appearance in the control region. The absence of the color band in the control region indicates that the results of the test are not valid, and, hence, a new device should be used to repeat the whole testing procedure [48,50,52].

### 4.11. RapiScan

**Figure 12:**
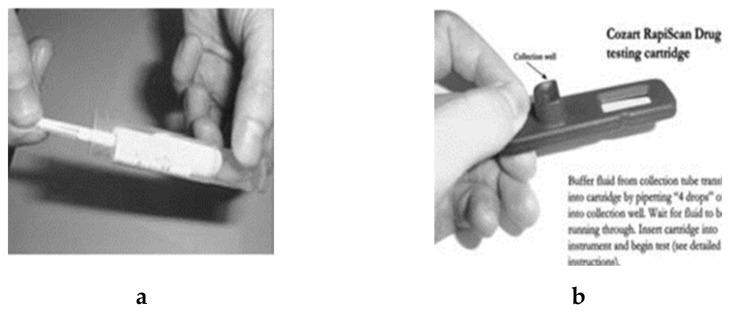

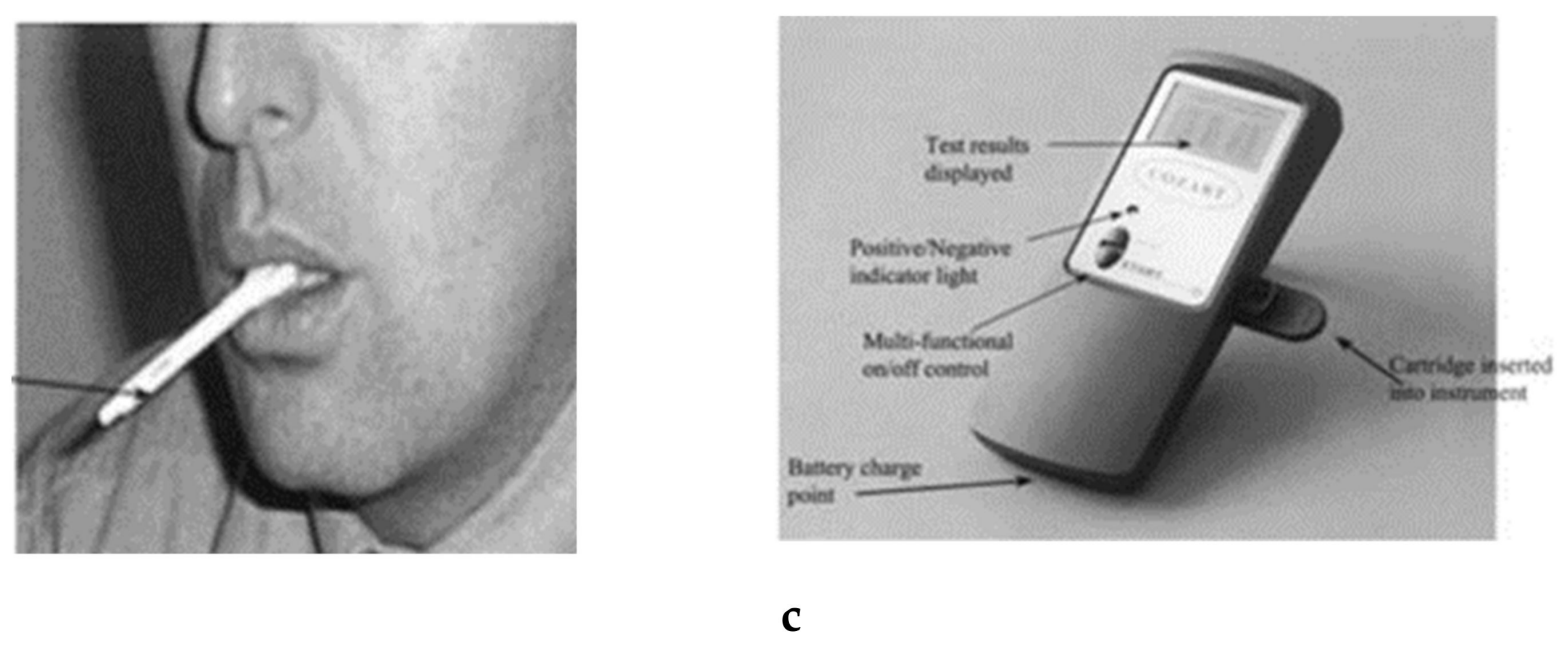
Cozart Rapiscan device. (**a**) The Cozart RapiScan Saliva Collection, (**b**) The Cozart RapiScan Drug testing cartridge, (**c**) The Cozart RapiScan Instrument with cartridge inserted (reproduced with permission from ASTM International [69]).

Another example of an oral fluid-based system of determining drug abuse is Cozart^®^ RapiScan (Figure 12) [69]. This detects six varying groups of drugs simultaneously: opiates, cannabinoids, benzodiazepines, methadone, amphetamine and cocaine from one single specimen [54]. The test system comprises three segments: the devices for collecting oral fluids, the drug kit, and the Cozart RapiScan device itself. The collection process is performed by placing a swab inside the mouth, which is used to collect the saliva sample. The collection process takes up a few minutes, and when enough sample has been obtained, a blue indicator shows at the end of the collector. An available test cartridge should be added, which evaluates the sample for the presence of drugs. The process can take up to a few minutes. The screening process takes place by an objective measuring device, which is used to screen the sample on site. The results are obtained on site in printed form within several minutes, so if the result appears as negative, the sample donor is allowed to leave [50]. This device weighs around 1000 g and costs around EUR 1000.

### 4.12. Sali•Chek™ System

**Figure 13:**
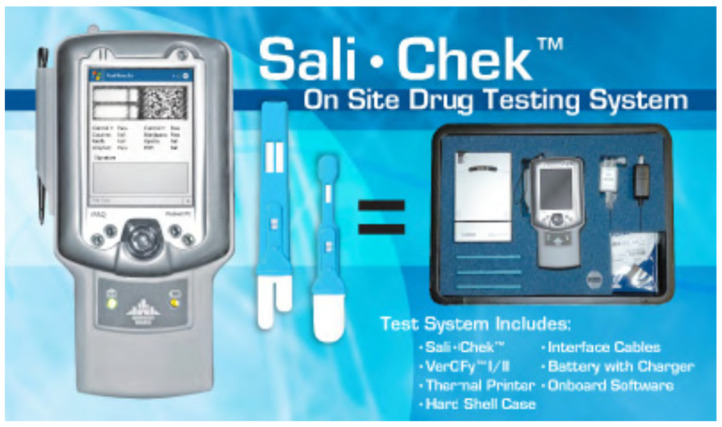
Sali Chek system [50].

Figure 13 represents a Sali•Chek™ System device. Drugs such as methamphetamines (meth), amphetamine, THC, phencyclidine, cocaine, and opiates, in a saliva sample, can be detected by this system. This system can also be stored at room temperature. A fork-shaped pad is used in order to obtain the saliva, which is then squeezed by the collector to collect approximately three to four drops of oral fluid. Specific drug kits (reagents) are used; 10 min are required to obtain the result, and 2 min are required for the reagents to dissolve. After the test starts, the validity of the test as well as the results should be deciphered within 10 to 20 min [48,52].

## 5. SalivaScreen

**Figure 14:**
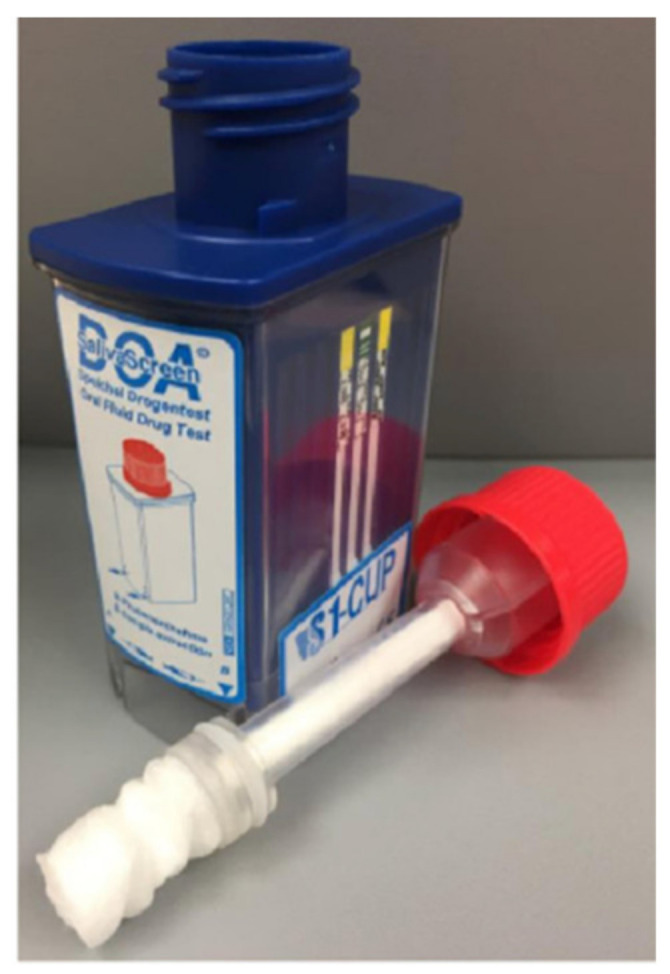
SalivaScreen device (reproduced with permission from Elsevier [72]).

SalivaScreen 5 (Figure 14) is a device that is able to check the presence of the following drugs: methamphetamines (meth), opiates, benzodiazepines, cocaine, and THC in a saliva sample. The system requires ambient temperature for storage. A swab is used to collect the oral fluid, and the swab is squeezed to collect approximately three to four drops of the oral fluid. This takes approximately two minutes to test, and after 10 min, the final results are obtained [48,62].

### 5.1. Smartclip Multidrug

**Figure 15:**
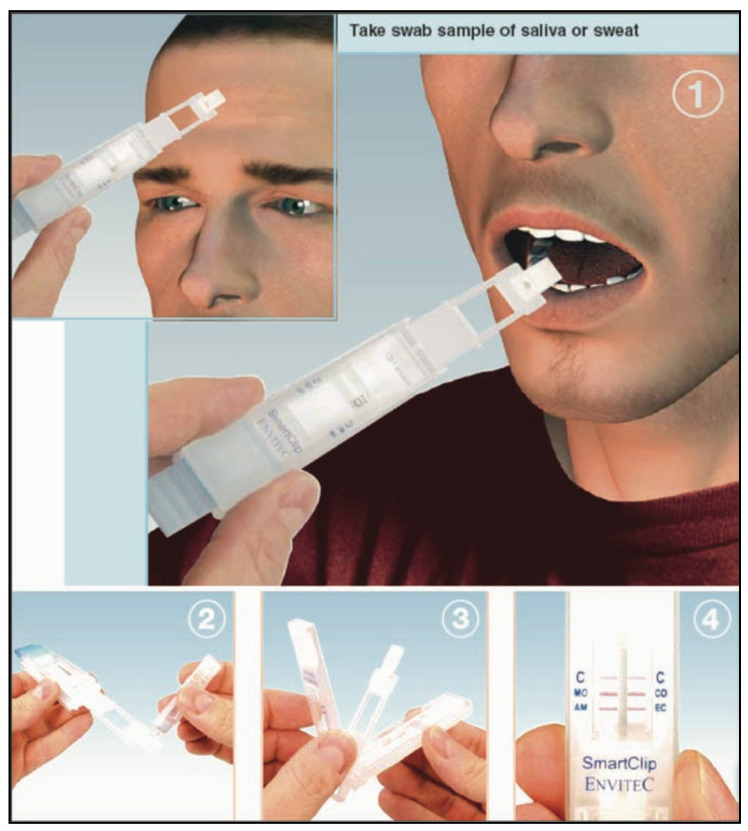
Smartclip device. (**1**) A swab of saliva or sweat is taken. (**2**) A buffer solution is added. (**3**) The system is closed. (**4**) The result can be read from indicator strips (reproduced with permission from Springer [51]).

Samples of a swab of saliva, sweat, and surfaces can be tested for drugs using Smartclip Multidrug, as displayed in Figure 15 [51]. Morphine, cocaine, methamphetamine, and amphetamine are the drugs that the device can detect precisely [57]. Some advantages of this system are that it is very easy to use and supplies very reliable results within an extremely short period of time. It takes about 60 s to generate proof of the drug’s absence, if the person who is tested has not consumed any drugs. In one single device, both the process of analysis and sample extraction occur. A change in color in the indicator strip indicates the test result, which can undergo further analysis using a scanner, or the results can be directly interpreted. SmartClip was tested in the German police’s daily work for a number of years and has proved to be reasonably reliable and practical [51].

### 5.2. Uplink/Drug Test

**Figure 16:**
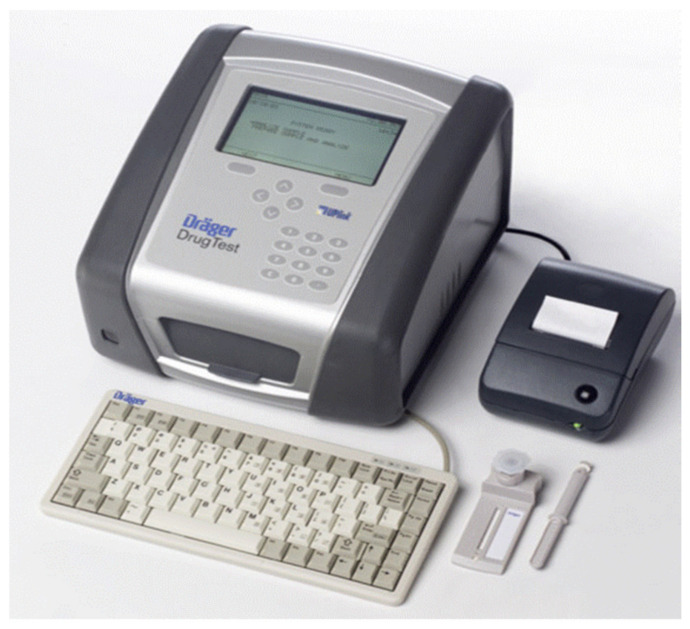
Uplink/Drug test device [50].

Drugs such as AMP, THC, OPI, COC and methamphetamine can be detected using the Uplink/Drug system. The collection of oral fluid samples here is conducted by a sponge. This device (Figure 16) functions as follows: (1) in the lower groove of the cassette, a buffer cartridge should be added, which can be performed by arranging the lower tabs on the cartridge, with notches on the cassette. (2) After placing the sample collection device into the buffer cartridge, the device can then start (if the sample collection device is pressed down with force, the device will set in its place and create a cracking sound). (3) This step involves turning the handle of the sample collection anticlockwise and removing the handle. (4) Approximately four minutes are required for incubation, and the buffer cartridge can then be closed. (5) The buffer cartridge must be kept moving in the clockwise direction. (6) This is continued until the upper groove aligns above the test cassette groove. (7) The buffer cartridge is pressed down until the wings of the cartridge meet the cassette edge. (8) The fluid is left to elute for about eight minutes. (9) The cassette is put into the analyzer and the ID of the sample entered. (10) Once the read cycle initiates, the door closes and the analyzer will indicate this. It takes approximately four minutes for the analyzer to report the result [50,58].

### 5.3. Fingerprint Drug Test

**Figure 17:**
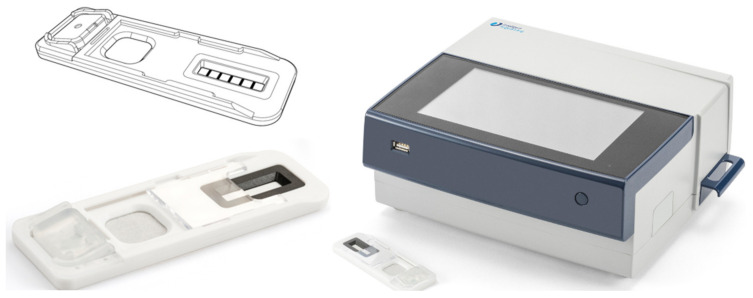
The Intelligent Fingerprint Drug Testing device (reproduced with permission from Oxford University Press [61]).

Intelligent Fingerprinting (Figure 17) developed an on-site testing device that uses fingerprint-based drug testing. Several types of drugs, including amphetamines, opiates, cocaine, methamphetamine, benzodiazepines, buprenorphine, methadone, and cannabis, can be detected by this fingerprint screening system. A small cartridge, which is tamper evident, is used to collect a sample of sweat from a fingerprint. An analysis is carried out by placing the cartridge containing the sample in the Intelligent Fingerprinting reader 1000. It takes around 10 min to receive the results, and the process of collecting the sample takes only about a few seconds [59,60,61]. The fingerprint test method uses a particle-based immunoassay. The finger is covered with a thin film of a labelled antibody and placed on a membrane containing immobilized ligand specifically selected to bind to the labelled antibody presenting in the fingerprint of the test subject, so that solution is deposited from the finger on the membrane indicating presence or absence of the substance being tested for [59,60,61].

### 5.4. Dried Blood Spot (DBS) Analysis

**Figure 18:**
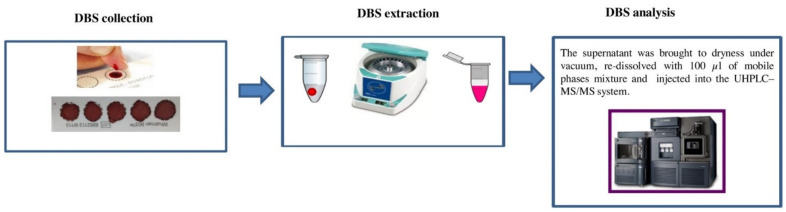
The procedure of dried blood spot analysis (reproduced with permission from Elsevier [36]).

Blood matrix can be easily collected using a finger prick; blood can be easily withdrawn on site by the police/the driver. Dried blood spots/dried serum spots could be used instead of venepuncture blood collection at the hospital, as it is cheaper and more accessible (Figure 18) [36].

## 6. Sample Collection, Storage and Pre-treatment

The collection step is a significant stage for the validity of a drug testing process. Various methods are used for oral fluid collection, including spitting, draining, suction or wiping the oral cavity with a swab [20]. Generally, spitting itself can be a sufficient stimulus to elicit flow; however, sometimes, due to the insufficient volume of the sample, the flow can be stimulated mechanically (e.g., chewing gum or an inert material) or chemically (e.g., citric acid or placing a sour sweet in the mouth), although this will reduce the collection time. It will also modify the pH of the oral fluid, which consequently affects the concentrations of drugs and metabolites. The storage of oral fluid samples should be at +4 °C and be examined as soon as possible. If longer storage is required, samples should be stored at −20 °C. Pre-treatment involves both liquid–liquid and solid-phase extraction in the case of a confirmation test [35].

One of the most significant samples of toxicological relevance is blood. This is because it offers distinctive benefits over other matrices due to the broad range of available analytical methodologies, the pharmacological interpretive value of the matrix, and the presence of the large number of reference data for detecting drug levels in both post-mortem and antemortem scenarios. A syringe or vacuum container (such as Vacutainer, Venoject) is used to obtain the antemortem blood from the arm’s antecubital region by venepuncture. In order to clean the site of blood collection, an antiseptic wipe is used before the sample collection, and to prevent any sort of contamination that could intervene with the alcohol assessment, antiseptic wipes, which are non-alcoholic, such as Betadine, are used. Although evacuated blood tubes are typically glass, plastic tubes have also been evaluated [36].

A plastic container consisting of sodium fluoride, which acts as a preservative, is used in order to collect a mid-stream urine specimen in the case of an antemortem scenario. In some instances, to prevent the adulteration of the sample, it might be essential to take certain precautions. A hypodermic syringe is inserted into the bladder to obtain the urine sample in post-mortem scenarios. To prevent any chance of contamination, abdominal wall puncture is avoided. It is similarly difficult to obtain urine on the roadside. Drug concentrations in urine, however, are not a productive method for establishing impairment, but help to determine any prior use of the drug. Table 3 represents known features of saliva, blood, and urine specimens at roadside drug testing [20].

### 6.1. Confirmatory Analysis Tests

Roadside testing is considered the first-line detection of illegal substances, and it is a presumptive test that needs to be followed by a confirmation test. In order to confirm the presence of an illegal drug, the samples should be sent to a testing laboratory for analysis [35]. Typically, this analysis requires a multi-step process by which the individual compounds are first separated by suitable techniques such as gas chromatography (GC) or liquid chromatography (LC), which are the most commonly used. Once the separation is performed, a combined detector such as a mass spectrometer (MS) is used to detect the desired compounds by comparing them against reference materials [62].

### 6.2. Gas Chromatography-Mass Spectrometry (GC-MS)

From the oral fluid, the drugs are extracted first before they are introduced to the GC-MS instrument. Derivatization may be required in order to make the sample volatile. The electron ionization (EI) technique is commonly used at 70 eV to ionize the molecules. For compound identification, unique fingerprints of ions are identified by the resulting mass to charge ratios along with their abundance. In order to analyze oral fluid drugs, several GC-MS methods were developed. Chemical ionization is used to produce fragments that are less extensive and more stable. Negative ion chemical ionization, however, creates fewer ions with high levels of sensitivity and lower selectivity. For confirmation analysis, GC-MS is usually operated in the selected ion monitoring mode, and deuterated internal standards are often used [35].

Generally, the ratios of three ions are measured. The primary advantages of the GC-MS instrument are affordability and robustness. Compared to the concentrations in blood, the majority of drug concentrations appear to be much less in oral fluid, so it requires further methods that are much more sensitive. Several studies have proven the efficiency of GC-MS in detecting various kinds of drugs such as cocaine [35,73,74].

### 6.3. Liquid Chromatography-Mass Spectrometry (LC-MS)

It is difficult to analyze certain drugs by GC-MS techniques, such as those that are present in low concentrations. LC-MS/MS has the advantage of being more sensitive than GC-MS. Hence, a lower-volume sample is required for LC-MS analysis. Furthermore, low recovery substances can be identified with this instrument. LC also allows the detection of both thermally labile and polar compounds. LC-MS techniques are particularly useful due to easy sample pre-treatment, as they do not have any complex derivation steps or procedures for cleaning the sample [75].

A screening of 32 substances using simple and rapid liquid–liquid extraction was performed by Oiestadt et al. LC-tandem mass spectrometry effectively tested and identified benzoylecgonine, even though the extraction recovery of benzoylecgonine was as little as 0.2%. For the analysis of 32 substances, the LC-MS/MS took about 20 min. Another study used the DYN-MRM-LC-MS/MS technique to detect 10 spiked drugs of abuse (terbutaline, ephedrine, methamphetamine, benzoylecgonine, clenbuterol, THC-d3, boldenone, stanozolol-d3, THC acid, and cannabidiol (THC)) in a single extract of a human hair sample [75].

The most widely used technique for ionization for drug analysis in LC-MS is electrospray ionization (ESI). Atmospheric pressure chemical ionization (APCI) is another process of ionization. There have been reports of several problems with the enhancement and suppression of ions; however, there can also be problems with APCI. Four sample preparation procedures, which are dilution, direct injection, solid-phase extraction, and protein precipitation, have been used to pre-treat samples and LC-ESI-MS/MS and LC-APCI-MS-MS undertook the analysis. Even though the matrix effect was seen in both types of ionization, ESI showed more vulnerability [35].

### 6.4. Direct Analysis in Real Time-Mass Spectrometry (DART-MS)

DART ion source was introduced to the field in 2005 [76]. This source utilizes a gas-phase ionization mechanism and it is suitable for the analysis of solids, liquids, and gases. No sample extraction process is required as the sample is directly analyzed on a surface such as paper or glass, which eventually reduces the analysis time. DART can be coupled with the MS detector or a high-resolution time-of-flight mass spectrometer to obtain a fast qualitative or quantitative analysis of an extensive range of substances [77].

DART-MS can provide an informative, sensitive, quick analytical screening technique compared to current techniques, which could permit rapid screening of unknown drugs that are commonly received in forensic laboratories [78]. DART has been used to detect various drugs of abuse, such as cathinone drug mixtures [79], heroin, LSD and other new psychoactive substances (NPSs) [80].

### 6.5. Liquid Chromatography-Time-of-Flight Mass Spectrometry (LC-TOF-MS)

TOF is a high-resolution technique that has recently emerged widely for high-throughput toxicological screening of unknown samples. A time-of-flight (TOF) or quadrupole time-of-flight (Q-TOF) analyzer commonly accompanies an LC/MS system. It provides high accuracy in mass measurements with detection limits up to the one part per million (ppm) level. It provides exceptional full-scan sensitivity and full screening of drugs, which can be achieved without predefined target analytes [81]. The advantage of high mass accuracy allows the detection of rare metabolites that have difficult-to-obtain standards by permitting the use of exact monoisotopic masses and isotopic configurations in the identification process. Moreover, screening any desired drugs in samples analyzed previously does not require re-analyzing as TOF-MS results can be reprocessed [82].

One drawback that has been noticed regarding this technique to drug abuse screening is the unavoidable false-positive data when only applying accurate mass and isotopic pattern matching for identification. However, checking fragmentation spectra alone is effective in reducing false-positive results. The success of LC-TOF-MS application in the drug toxicology field has been accomplished by Pelander, Kolmonen, Ojanperä, and Gergov et al. [81]. Groups of drug standards, such as benzodiazepines, opiates, stimulants, antidepressants, synthetic cannabinoids, and cathinone have been analyzed using LC–TOF-MS [83].

### 6.6. Ambient Ion Sources for Direct Forensic Analysis

To meet the needs of the forensic and security communities, fieldable mass spectrometers based on membrane inlet systems and hybrid gas chromatography systems have been developed and commercialized. More recently, developed ambient ionization mass spectrometry methods can eliminate the time, equipment, and expertise associated with sample preparation and so are especially appealing for on-site analysis. The development of fieldable mass spectrometry systems, with emphasis on commercially available systems, could be used for the on-site analysis of seized drugs, explosives, chemical warfare agents and other compounds of interest [37].

## 7. Conclusions

In conclusion, drunk and drug driving is a big problem around the world [1,2]. There are a number of debates about the types and sensitivities of roadside testing devices; current technologies can successfully be implemented on the roadside and can detect significant numbers of impaired drivers. Sample collection and pre-treatment are used to make the extract ready for use in the testing devices. There are many roadside testing devices available for the quick screening of samples. This article will help law enforcement agencies to evaluate all of these devices in one place and decide which device is suitable for their needs. There are many pros and cons of the devices discussed in this paper. Screening is followed by new and emerging confirmatory devices to confirm the presence of illicit drugs. These relatively new data on drug driving clearly indicate the need for enforcement, education, prevention, and, most importantly, the need for more research into human behavior to better inform responses and interventions.

## Figures and Tables

**Table 1:** On-site systems are employed for the determination of various types of drugs.

Device	Principle of Operation	Tested Drugs/Drug Classes	Cutoff	Time of Analysis	Body Sample Used	Complexity/ Difficulty	Reference
Securetec Drugwipe	Immunological rapid screening test.	Cannabis, Opiates, Cocaine, Amphetamines, Methamphetamines (MDMA, ecstasy), Benzodiazepines and Ketamine.	Amphetamine: 50 ng/mL Methamphetamine: 25 ng/mL MDMA: 25 ng/mL Cocaine: 30 ng/mL Opiates: 10 ng/mL Cannabis: 30 ng/mL	5 min	saliva, sweat, surface	easy	[3,20,40,41,42]
Draeger DrugTest 5000 Analyzer	Colorimetric technique.	Amphetamines, Benzodiazepine, THC, Cocaine, Methamphetamines, Opiates and Methadone.	Amphetamine: 50 ng/mL Methamphetamine: 35 ng/mL Cocaine: 20 ng/mL Opiates: 20 ng/mL Cannabis: 25 ng/mL Benzodiazepines: 15 ng/mL Cannabis: 5 ng/mL	It depends on the test kit (normally less than 9 min)	Saliva/surfaces	moderately easy	[43,44,45]
Alere DDS2	A lateral flow immunoassay device.	Amphetamine, Benzodiazepines, Cannabis, Cocaine, Methamphetamine, and Opiates.	Cannabis: 25 ng/mL Cocaine: 30 ng/mL Amphetamine: 50 ng/mL Methamphetamine: 50 ng/mL Benzodiazepines: 20 ng/mL Opiates: 40 ng/mL	within 5 min	saliva	easy	[46,47]
Mavand Rapid STAT	A lateral flow immunoassay device.	Amphetamines, Benzodiazepines, Cocaine, Methamphetamines, MDMA, Opiates and Marijuana (THC).	Amphetamine: 25 ng/mL Benzodiazepines: 25 ng/mL Cocaine: 12 ng/mL Methamphetamine: 25 ng/mL MDMA: 50 ng/mL Opiates: 25 ng/mL Cannabis: 15 ng/mL	Results are ready within around 7 to 12 min	saliva	less easy	[42,45]
iScreen Test Device	A lateral flow chromatography immunoassay.	Cocaine (COC), Marijuana (THC), Methamphetamine (Meth), Amphetamine (AMP), Opiate (OPI), and Phencyclidine (PCP).	THC: 100 ng/mL Cocaine: 20 ng/mL Methamphetamine: 50 ng/mL Amphetamine: 50 ng/mL Phencyclidine: 10 ng/mL Opiates: 40 ng/mL	Within around 10 min	Saliva/surfaces	easy	[48]
OraLab	A lateral flow immunoassay device.	Amphetamines, Methamphetamine, Cocaine, Opiates, Cannabis (THC), and Phencyclidine (PCP).	Amphetamine: 50 ng/mL Cocaine: 20 ng/mL Methamphetamine 50 ng/mL PCP: 10 ng/mL Opiates: 40 ng/mL Cannabis: 50 ng/mL	Results will be obtained in 10 to 15 min.	saliva	moderately easy	[45,46,49,50,51]
AconLabs Oral Fluid Drug Screen	A lateral flow immunoassay technique.	Cocaine, Cannabis, Amphetamines, Methamphetamines, Opiates, and Phencyclidine.	THC: 12 ng/mL Cocaine: 20 ng/mL Amphetamine: 50 ng/mL Methamphetamine: 50 ng/mL Phencyclidine: 10 ng/mL Opiate: 40 ng/mL	Within about 10 min	saliva	moderately easy	[48,50]
OralStat	Lateral flow immunoassay testing.	Including but not limited to: Benzodiazepines, Amphetamines, Cocaine, Methadone, Methamphetamines, Opiates, Phencyclidine, Barbiturates, Buprenorphine, MDMA, Oxycodone, Tricyclic antidepressants and THC.	Amphetamine: 25 ng/mL Methamphetamine: 25 ng/mL Benzodiazepines: 25 ng/mL Cocaine: 12 ng/mL Methadone: 25 ng/mL Opiate: 20 ng/mL Phencyclidine: 5 ng/mL THC: 25 ng/mL	2 to 4 min	saliva	less easy	[50]
Oratect II	Immunoassay technique.	Amphetamine, Marijuana, Cocaine, Benzodiazepines, Opiates, Phencyclidine and Methamphetamine	Amphetamine: 25 ng/mL Methamphetamine: 25 ng/mL MDMA: 25 ng/mL Cocaine: 20 ng/mL Opiates: 10 ng/mL Cannabis: 40 ng/mL Benzodiazepines: 5 ng/mL	8 min	saliva	easy	[48,50,52]
RapiScan	Immunoassay technique.	Opiates, Cannabinoids, Benzodiazepines, Methadone, Amphetamine, Methamphetamine and Cocaine.	Opiates: 45 ng/mL THC: 150 ng/mL Benzodiazepines: 60 ng/mL Methadone: 15 ng/mL Amphetamine: 45 ng/mL Methamphetamine: 45 ng/mL Cocaine. 30 ng/mL	few minutes	saliva	moderately easy	[48,50,51]
Sali•Chek™ System	Immunoassay technique.	Methamphetamines, Amphetamine, THC, Phencyclidine, Cocaine and Opiates.	THC: 12 ng/mL Cocaine. 20 ng/mL Opiates: 40 ng/mL Phencyclidine: 10 ng/mL Amphetamine: 50 ng/mL Methamphetamine: 50 ng/mL	10 to 20 min	saliva	less easy	[50,51,52,53,54,55]
SalivaScreen 5	Immunoassay technique.	THC, Cocaine, Opiates, Methadone, Amphetamine and Methamphetamine.	THC: 2 ng/mL Cocaine. 30 ng/mL Morphine: 30 ng/mL Methadone: 30 ng/mL Amphetamine: 50 ng/mL Methamphetamine: 50 ng/mL	10 min	saliva	easy	[53,54,55,56,57,58,59,60,61,62]
Smartclip Multidrug	Immunoassay technique.	Amphetamine, Methamphetamine, Cocaine and Morphine.	Amphetamine: 50 ng/mL (saliva)/20 ng/mL (sweat) Methamphetamine: 100 ng/mL (saliva)/40 ng/mL (sweat) Cocaine: 20 ng/mL (saliva)/ 8 ng/mL (sweat) Morphine: 40 ng/mL (saliva)/ 16 ng/mL (sweat)	60 s	saliva, sweat	easy	[51,52,53,54,55,56,57]
Uplink/Drug Test	Immunoassay technique using up-converting phosphor technology.	Amphetamine, Methamphetamine, THC, Cocaine and opiates.	THC: 20 ng/mL Cocaine. 5 ng/mL Opiates: 5 ng/mL Methamphetamine: 10 ng/mL Amphetamine: 10 ng/mL	4 min	saliva	moderately easy	[58]
Fingerprint Drug Test	Particle-based immunoassay.	Amphetamines, Opiates, Cocaine, Methamphetamine, Benzodiazepines (BNZ), Buprenorphine, Methadone and Cannabis.	THC: 190 pg/fingerprint BNZ: 90 pg/fingerprint Morphine: 68 pg/fingerprint Amphetamine: 80 pg/fingerprint	10 min	fingerprint-based	easy	[59,60,61]

**Table 2:** Some of the advantages and disadvantages of on-site drug systems.

Device	Advantages	Disadvantages	References
Securetec-DrugWipe	Fast and easy sample collection.	Devices must be kept still during analysis.	[63]
	Detects illegal drugs on surfaces, skin and saliva.	Not sensitive and accurate enough for cannabis.	
	User-friendly	Difficult to provide saliva samples.	
	Fits in any pocket.	If a subject eats, drinks or smokes within 10 minutes of the test being performed, the results can be compromised.	
		A negative result may not necessarily indicate a drug-free specimen. Drugs may be present in the specimen below the cut-off level of the assay.	
Draeger DrugTest 5000 Analyzer	Reproducibility of the results.	Difficult to provide saliva samples.	[64,65]
	Safe operation and hygienic.	If a subject eats, drinks or smokes within 10 minutes of the test being performed, results can be compromised.	
	The analyzer can save up to 500 individual measurements.	Results can be affected by the environment (e.g., temperature).	
	Cost-effective	A negative result may not necessarily indicate a drug-free specimen. Drug may be present in the specimen below the cut-off level of the assay.	
	Detects illegal drugs on surfaces and saliva.		
Alere DDS2	Robust	Difficult to provide saliva samples.	[3,20,65]
	Easy to use.	Results can be affected by the environment (e.g., temperature and humidity).	
	Simple collection process.	The device must be kept still during analysis.	
	The analyzer can store 10,000 results.	The time required for collecting the sample is quite long.	
		If a subject eats, drinks or smokes within 10 minutes of the test being performed, results can be compromised.	
		A negative result may not necessarily indicate a drug-free specimen. Drugs may be present in the specimen below the cut-off level of the assay.	
Mavand Rapid STAT	Fast sample collection.	The test must always be allowed to warm up to room temperature before any testing is conducted (results affected by environment temperature).	[45,66,67]
	Easy to use because of the One-Hand-Clip-System.	If a subject eats, drinks or smokes within 10 minutes of the test being performed, results can be compromised.	
	Rapid STAT Saliva Drug Test achieves 80 to 90% sensitivity in the detection of THC.	A negative result may not necessarily indicate a drug-free specimen. Drugs may be present in the specimen below the cut-off level of the assay.	
	Cost-effective		
iScreen Test Device	Detects illegal drugs on surfaces and saliva.	Difficult to provide saliva samples.	[48,68]
	Simple procedure.	For invalid results, the test needs to be repeated using a new device.	
	Easy sample collection.	Results can be affected by the environment (e.g., temperature).	
	User-friendly	A negative result may not necessarily indicate a drug-free specimen. Drugs may be present in the specimen below the cut-off level of the assay.	
	Low cost.		
OraLab	Good specificity.	Time-consuming	[49]
	Simple operation.	Difficult to provide saliva samples.	
	Safe operation and hygienic.	If a subject eats, drinks or smokes within 10 minutes of the test being performed, results can be compromised.	
	Easy Interpretation of results.	Low sensitivity for cocaine and THC.	
		Results can be affected by environment temperature, so if the foil pouch containing the OraLab ^®^ 6 profile card is damaged (e.g., a hole or tear), the device cannot be used.	
AconLabs Oral Fluid Drug Screen	Safe operation and hygienic.	Difficult to provide saliva samples.	[48]
	Easy sample collection.	If a subject eats, drinks or smokes within 10 minutes of the test being performed, results can be compromised.	
	Simple procedure.		
	Easy Interpretation of results.		
OralStat	Fast	Difficult to provide saliva samples.	[50]
	Safe operation and hygienic.	If a subject eats, drinks, or smokes within 10 minutes of the test being performed, results can be compromised.	
	Rapid test result.		
Oratect^®^ II	Easy to use.	If a subject eats, drinks or smokes within 10 minutes of the test being performed, results can be compromised.	[48]
	One-step process.	A negative result may not necessarily indicate a drug-free specimen. Drugs may be present in the specimen below the cut-off level of the assay.	
	User-friendly.	Low sensitivity for amphetamine.	
	Easy Interpretation of results.		
RapiScan	Fast	Difficult to provide saliva samples.	[69]
	Sensitive, specific, and reliable.	If a subject eats, drinks or smokes within 10 minutes of the test being performed, results can be compromised.	
		Results can be affected by environmental temperature.	
Sali•Chek™ System	Safe operation and hygienic.	Time-consuming	[50]
	Robust	If a subject eats, drinks, or smokes within 10 minutes of the test being performed, results can be compromised.	
		Results can be affected by environmental temperature.	
SalivaScreen 5	The lowest THC testing sensitivity available.	Difficult to provide saliva samples.	[70]
	Cost-effective	If a subject eats, drinks, or smokes within 10 minutes of the test being performed, results can be compromised.	
	Simple and easy to use.	Results can be affected by environmental temperature.	
		The oral fluid samples must be collected with the collector provided with the kit (no other collection devices can be used).	
Smartclip Multidrug	Fast test result.	Difficult to provide saliva samples.	[51]
	Easy operation.	If a subject eats, drinks or smokes within 10 minutes of the test being performed, results can be compromised.	
	Detects illegal drugs on skin and saliva.	Results can be affected by environmental temperature.	
	Hygienic and safe disposable system.		
Uplink/Drug Test	Rapid test result.	Difficult to provide saliva samples.	[50]
	Robust	If a subject eats, drinks or smokes within 10 minutes of the test being performed, results can be compromised.	
		Multi-steps required for operation (not easy).	
Fingerprint Drug Test	Simple and easy to operate.	Run time quite long.	[71]
	Safer, socially distanced drug testing.	It does not distinguish either positive drugs were ingested or as a result of recent contact with the drug.	
	Cost-effective		

**Table 3:** Characteristics of oral fluid, blood, and urine relating to roadside drug testing.

Specimen	Collection	Collection Time	Sample Integrity	Ability in Determining the Presence of Drugs	
Oral Fluid	Can be easily collected by any trained officer at roadside.	Takes up to 10 min.	Not easily adulterated if the collection is carefully observed.		Able to detect.
Blood	Not practical and requires a nurse/doctor to collect at a clinic/hospital.	Depends on the availability of a nurse/doctor (must be taken within 3 h).	Not easily adulterated.		Able to detect.
Urine	Not practical and requires a nurse/doctor to collect at a clinic/hospital.	Depends on the ability of accused to give a sample (must be taken within 3 h).	Not easily adulterated if the collection is carefully observed.		Able to detect.
